# Well-differentiated gastroenteropancreatic G3 NET: findings from a large single centre cohort

**DOI:** 10.1038/s41598-021-97247-x

**Published:** 2021-09-09

**Authors:** K. Lithgow, H. Venkataraman, S. Hughes, H. Shah, J. Kemp-Blake, S. Vickrage, S. Smith, S. Humphries, M. Elshafie, P. Taniere, S. Diaz-Cano, B. V. M. Dasari, M. Almond, S. Ford, J. Ayuk, S. Shetty, T. Shah, I. Geh

**Affiliations:** 1 Division of Endocrinology, Department of Medicine, Cumming School of Medicine, 1820 Richmond Rd SW, Calgary, AB T2T 5C7 Canada; 2 Department of Endocrinology, University Hospitals Birmingham NHS Foundation Trust, Birmingham, UK; 3 Department of Radiology, University Hospitals Birmingham NHS Foundation Trust, Birmingham, UK; 4 Department of Liver Medicine, University Hospitals Birmingham NHS Foundation Trust, Birmingham, UK; 5 Department of Pathology, University Hospitals Birmingham NHS Foundation Trust, Birmingham, UK; 6 Department of Liver Surgery, University Hospitals Birmingham NHS Foundation Trust, Birmingham, UK; 7 Department of General Surgery, University Hospitals Birmingham NHS Foundation Trust, Birmingham, UK; 8 Department of Oncology, University Hospitals Birmingham NHS Foundation Trust, Birmingham, UK

**Keywords:** Endocrine cancer, Neuroendocrine cancer, Gastrointestinal cancer

## Abstract

Neuroendocrine neoplasms are known to have heterogeneous biological behavior. G3 neuroendocrine tumours (NET G3) are characterized by well-differentiated morphology and Ki67 > 20%. The prognosis of this disease is understood to be intermediate between NET G2 and neuroendocrine carcinoma (NEC). Clinical management of NET G3 is challenging due to limited data to inform treatment strategies. We describe clinical characteristics, treatment, and outcomes in a large single centre cohort of patients with gastroenteropancreatic NET G3. Data was reviewed from 26 cases managed at Queen Elizabeth Hospital, Birmingham, UK, from 2012 to 2019. Most commonly the site of the primary tumour was unknown and majority of cases with identifiable primaries originated in the GI tract. Majority of cases demonstrated somatostatin receptor avidity. Median Ki67 was 30%, and most cases had stage IV disease at diagnosis. Treatment options included surgery, somatostatin analogs (SSA), and chemotherapy with either platinum-based or temozolomide-based regimens. Estimated progression free survival was 4 months following initiation of SSA and 3 months following initiation of chemotherapy. Disease control was observed following treatment in 5/11 patients treated with chemotherapy. Estimated median survival was 19 months; estimated 1 year survival was 60% and estimated 2 year survival was 13%. NET G3 is a heterogeneous group of tumours and patients which commonly have advanced disease at presentation. Prognosis is typically poor, though select cases may respond to treatment with SSA and/or chemotherapy. Further study is needed to compare efficacy of different treatment strategies for this disease.

## Introduction

Neuroendocrine neoplasms (NEN) are a heterogenous group of rare tumours that secrete peptides and neuroamines^1,2^. The WHO 2019 classification for Digestive Diseases categorizes these tumours based on differentiation (well differentiated vs. poorly differentiated), mitotic rate, and Ki67 index^3^. Grade 3 NET have been shown to be clinically distinct from NEC, with higher rates of avidity on somatostatin receptor scintigraphy (SRS), lower Ki67, poorer responses to platinum based chemotherapy, and longer survival^4–8^.

The estimated prevalence of G3 NET amongst all gastroenteropancreatic (GEP) NEN is 5.6 to 8%^4–6^. Mean age at presentation is in the sixth decade^5,6,8^. Primary tumours are mostly located in the pancreas, stomach, small bowel, and colon, with the pancreas being the most common site^4,8^. Only 5 to 25% of G3 NET are functional^5,6,8^ and 87 to 92% show SRS positivity on imaging^6,8,9^. Across different series, median Ki67 ranges from 30 to 40%^6–8^. Survival is variable, with median overall survival ranging from 41 to 99 months^5–8^. The prognosis of G3 NET is thought to be intermediate between G2 NET and G3 NEC^10,11^.

There are no established treatment guidelines for G3 NET^10^. Though historically platinum-based chemotherapy has been used first-line, results from multiple cohort studies suggest that G3 NET do not respond well to this treatment, with lower objective response rates and progression-free survival compared with G3 NEC^8,10,12^. Recently, temozolomide-based chemotherapy, such as CAPTEM (capecitabine and temozolomide) have shown more promising results in the treatment of G3 NET^10,13–15^. Other proposed treatment options include sunitinib and everolimus^10,16–18^ dependant on local funding practises. Given that majority of these tumours are SRS positive, peptide receptor radionuclide therapy (PRRT) has been implicated as a treatment strategy, though funding for this treatment will be limited by lack of clinical trial evidence of efficacy and cost-effectiveness. Two recent cohort studies reported 58^19^ and 11^20^ cases of G3 NET treated with PRRT. Carlsen et al. reported promising results with partial response observed in 42% and stable disease in 51% of G3 NET cases^19^. Recently, immunotherapies have been explored for treatment of NEN, however, their efficacy for this disease, and in particular for the G3 NET subgroup, has not been established^10^.

Most existing data on G3 NET are derived from small retrospective cohort studies^5–8^, therefore evidence to predict disease behavior and to guide treatment decisions for these tumours is limited^10^. We aimed to contribute to the existing body of evidence on G3 NET by describing clinical characteristics, treatment, and outcomes in a large single centre cohort.

## Patients and methods

We retrospectively reviewed the records from all patients registered in the NET database at Queen Elizabeth Hospital between 2012 and 2019. This study was conducted in accordance with published guidelines for retrospective chart review^21^. The study was registered and approved as an audit by our hospital’s Clinical Audits and Registries Management Service (CARMS), therefore review by an independent ethics committee was not required and the requirement for informed consent was waived. To be eligible for inclusion, all cases were required to be classified as G3 NET based on both well-differentiated tumour morphology and Ki67 > 20%. Given the changes in classification of G3 NEN in recent years, dedicated review of all pathology reports was performed at the time of data collection in accordance with most recent classification of G3 NET^3^. Tumours with poorly-differentiated morphology, or mixed well differentiated and poorly differentiated morphology were excluded. All referrals are discussed at the specialist NET multidisciplinary team meeting (MDT) before entry into the clinical pathway. The core NET-MDT consists of a radiologist, nuclear medicine radiologist, histopathologist, specialist nurses, hepatologist, oncologist, endocrinologist, liver and GI surgeons. Initial diagnostic work-up includes biochemistry (chromogranin A and B, urine 5 HIAA, fasting gut hormones) cross sectional imaging, and nuclear imaging as deemed appropriate by the treating clinician. Tissue diagnosis is obtained in all cases, including dedicated review by a histopathologist with expertise in NEN.

Response to treatment was classified as complete response (CR) partial response (PR), stable disease (SD), or progressive disease (PD) based on dedicated radiology review of all post treatment cross-sectional imaging in accordance with RECIST 1.1 criteria^22^. Progression free survival was determined based on time from treatment initiation until time where PD was detected on imaging (or time until death if the patient did not survive long enough to undergo imaging). Disease control was defined by the total number of cases showing SD, PR, and CR. Total length of survival was determined based on time from initial review at NET-MDT until time of death or time of last clinical follow-up. Kaplan–Meier estimates were used to calculate survival and progression free survival. We employed the Cox proportional hazard model for multivariate analyses and generated hazard ratios with corresponding 95% CIs. Statistical analysis was performed using SPSS version 26.

### Ethics approval

The study was registered and approved as an audit by our hospital’s Clinical Audits and Registries Management Service (CARMS), therefore review by an independent ethics committee was not required.

## Results

Initial interrogation of the database for patients with grade 3 tumours yielded 110 potentially eligible cases. After dedicated review of clinical and pathological characteristics, 84 cases were excluded due to differentiation status (poorly differentiated NEC) or non-GEP primary. 26 cases were included in the final analysis (Table 1). 46% (12/26) were female. Median age at diagnosis was 67.5 (range 23–83) years. 50% (13/26) had no detectable primary tumour and were classified as unknown primary but were suspected to be gastroenteropancreatic in origin due to the presence of liver metastases in 12/13 at diagnosis. 15% (4/26) had pancreatic primary, 34% (9/26) had primaries in the gastrointestinal tract (n = 3 small bowel, n = 2 colon, n = 2 gastric, n = 1 rectal, n = 1 gallbladder). 8% (2/26) had stage II disease, 12% (3/26) had stage III disease, and 80% (21/26) had stage IV disease with distance metastases at diagnosis. Liver metastases were seen in 95% (20/21) of patients with stage IV disease.

**Table 1:** Baseline characteristics of n = 26 cases.

Characteristic	Median (range)/proportion
Age at diagnosis	67.5 (23–83)
Sex	46% (12/26) female
Primary	50% (13/26) unknown
	15% (4/26) pancreatic
	34% (9/26) gastrointestinal
	n = 3 small bowel
	n = 2 colon
	n = 2 gastric
	n = 1 rectal
	n = 1 gallbladder
**Stage at diagnosis**	
II	8% (2/26)
III	12% (3/26)
IV	80% (21/26)
Surgery of primary	19% (5/26)
Elevated urine 5HIAA	47% (8/17)
Carcinoid syndrome	4% (1/26)
Elevated CgA	68% (17/25)
Elevated CgB	52% (13/25)
SSR avidity	56% (9/16) avid
	31% (5/16) non avid
	13% (2/16) mixed
Ki67	30% (20–70%)
**Ki67 distribution**	
20–29%	27% (7/26)
30–39%	31% (8/26)
40–49%	15% (4/26)
50–59%	12% (3/26)
60–69%	12% (3/26)
> 70%	4%(1/26)

SSR+: somatostatin receptor avid; SSA: somatostatin analog; PD: progressive disease; SD: stable disease.

Urine 5-HIAA was measured in 17 patients and found to be elevated in 47% (8/17) patients (n = 4 unknown primary, n = 3 small bowel, n = 1 pancreas). Carcinoid syndrome (defined as patient reported history of increased frequency of bowel movements and flushing with elevated 5-HIAA) was present in 4% (1/26) (unknown primary). Chromogranin (Cg) A was elevated in 68% (17/25), CgB was in 52% (13/25) and both CgA and CgB in 48% (12/25). Fasting gut hormones (gastrin, glucagon, pancreatic polypeptide, VIP, and somatostatin) were measured in 50% (13/26) cases (n = 8 unknown, n = 2 pancreatic, n = 1 rectal, n = 1 small bowel, n = 1 gallbladder primary). Gastrin level was elevated (100 times upper limited normal) in 1 case (unknown primary) which clinically was in keeping with metastatic gastrinoma. Glucagon was elevated (3.5 times upper limit normal) in one case (gallbladder primary). Fasting gut hormones were normal in the remaining 11/13 cases.

Functional somatostatin receptor (SSR) imaging was performed in 62% (16/26); 13/26 with octreotide scan in 3/26 with gallium-68 DOTATOC PET. Of these, 56% (9/16) were SSR avid, 31% (5/16) were non SSR avid, and 13% (2/16) showed a mixture of SSR avid and non SSR avid lesions. FDG-PET scan was performed in 3/26 (12%) (n = 1 with non-avid lesions on gallium-68 DOTATOC PET and n = 2 which did not have functional SSR imaging performed); all three demonstrated FDG avid disease.

Median Ki67 was 30% (range 20–70%). 15% (4/26) had Ki67 ≥ 55%. For pathological diagnosis, tissue from the primary tumour was used in 23% (n = 6); the remaining cases were diagnosed based on liver biopsy (n = 19) and bone biopsy (n = 1). Amongst the 13 cases with unknown site of primary tumour, diagnosis was made by liver biopsy in 12 cases and bone biopsy in one case.

19% (5/26) had surgery for the primary tumour, (n = 2 pancreas, n = 2 colon, n = 1 gastric), 2 of which had R0 resection. 34% (9/26) received somatostatin-analogues (SSA). 8/9 cases were SSR avid and one case had a mixture of SSR avid and non SSR avid lesions on functional imaging. The case (n = 1) with carcinoid syndrome had documented improvement in symptoms (self-reported reduced frequency of bowel movements and/or improvement in flushing) following treatment with SSA. 6/9 cases showed progressive disease following initiation of SSA. 3/9 cases showed stable disease followed by progression at 9, 9, and 16 months. 3/9 cases initially treated with SSA went on to receive chemotherapy. Estimated median progression free survival (PFS) following initiation of SSA was 4.0 months (95% CI 0.0–8.4 months). Details of cases treated with SSA are shown in Table 2.

**Table 2:** Outcomes following treatment with SSA and chemotherapy.

Treatment	Proportion	Disease outcome	Estimated median PFS
SSA	34% (9/26)	PD n = 6	4 months (95% CI 0.0–8.4 )
		SD n = 3	
**Chemotherapy**	43% (11/26)		3.0 months (95% CI 0.0–6.2)
CAPTEM	n = 8	PD n = 4	
		SD n = 2	
		PR n = 2	
Carboplatin + etoposide	n = 3	PD n = 2	
		PR n = 1	

SSA: somatostatin analog; PD: progressive disease; SD: stable disease; PR: partial response; OS: overall survival; PFS: progression free survival; CAPTEM: capecitabine and temozolomide.

43% (11/26) received chemotherapy; eight received chemotherapy alone and three received SSA with chemotherapy. Chemotherapy regimens were CAPTEM in 73% (n = 8) and carboplatin plus etoposide in 27% (n = 3). Estimated median progression free survival in the chemotherapy group was 3.0 months (95% CI 0.0–6.2); there was no significant difference (p = 0.336) in survival between cases receiving platinum based chemotherapy (n = 3) and CAPTEM (n = 8). 3 cases had partial response with shrinkage of liver metastases on follow-up imaging, and went on to have PD at 13, 17, and 22 months. Two patients had stable disease following chemotherapy initiation and then went on to have progressive disease at 5 and 10 months. 6 patients had progressive disease within 3 months of chemotherapy initiation. Amongst cases that received carboplatin plus etoposide (n = 3), one case showed disease control with PR. Of the cases that received CAPTEM (n = 8), 4 cases showed disease control with PR (n = 3) or SD (n = 1). Details of cases treated with chemotherapy are shown in Table 2.

No cases underwent treatment with 177-lutetium peptide receptor radionuclide therapy (PRRT). 12% (3/26) cases did not receive any surgical or medical treatment aside from palliative care due to advanced disease and poor performance status at baseline; median survival of these cases was 2 months (range 1 to 3 months).

**Figure 1:**
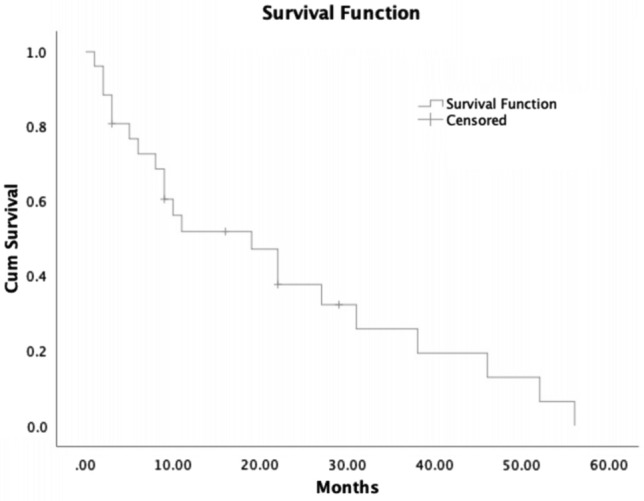
Overall survival in cases of G3 NET.

**Figure 2:**
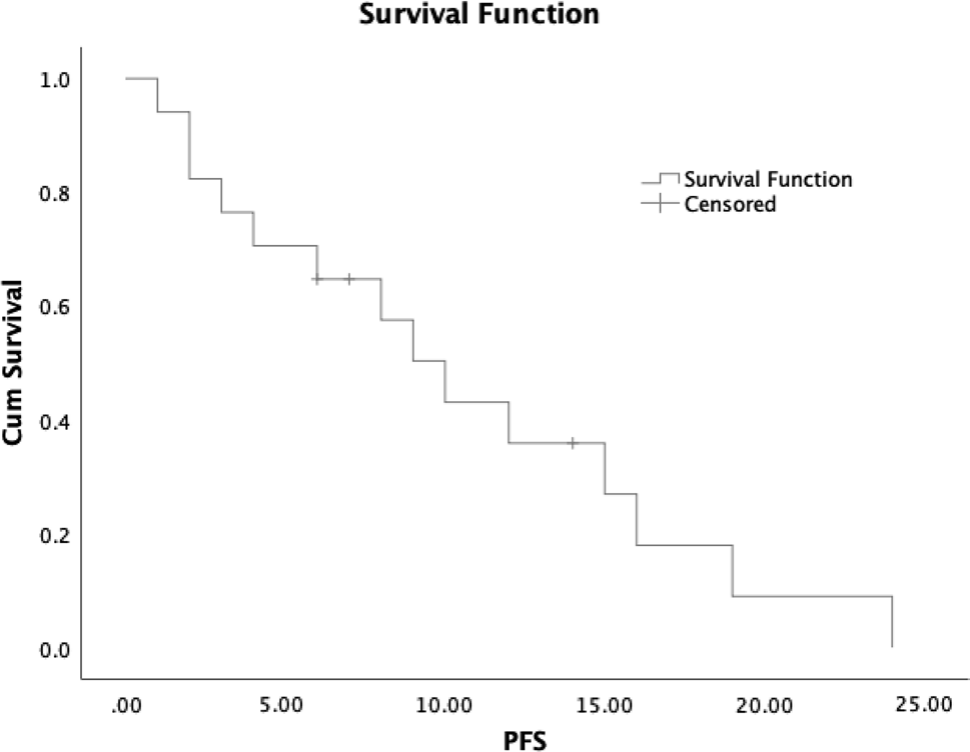
Progression free survival in cases of G3 NET.

Median follow-up was 10.5 months (range 1 to 56 months). At time of last follow-up, 19% (5/26) remained alive. Estimated median overall survival was 19 months (95% CI 6.0–32.0); estimated 1 year survival was 60% (95% CI 0.4–0.8) and estimated 2 year survival was 13% ((95% CI 0.0–0.3) (Fig. 1). Estimated median progression free survival was 10 months (95% CI 6.5–13.5) (Fig. 2) Estimated median overall survival was 22 months for cases with stage I–III disease and 11 months for cases with stage IV disease at presentation (p = 0.496) (Supplementary Figure). We employed Cox proportional hazards using primary disease site, Ki67 ≥ 55%, and stage IV disease at presentation as covariates (Table 3).

**Table 3:** Results from Cox proportional hazard model.

Variable	HR (95% CI)	p-value
Ki67 ≥ 55%	0.255 (0.247–6.737)	0.763
Stage IV disease	1.052 (0.209–39.186)	0.430
**Primary**		
Unknown	Reference	
Gastric	− 0.644 (0.040–6.8530)	0.623
Small Bowel	2.307 (0.317–317.717)	0.191
Pancreatic	− 0.566 (0.032–10.085)	0.700
Colon	− 0.284 (0.045–12.694)	0.844
Rectal	− 13.420 (0.000–∞)	0.987
Gallbladder	− 13.209 (0.000–∞)	0.991

## Discussion

We describe clinical and follow-up data for a large cohort of G3 NET. Previously published series reporting on treatment and outcomes for G3 NET are shown in Table 4. Median overall survival of 19 months in our cohort was shorter when compared with most previously published series of G3 NET^5,6,8,9,13–15,23–25^ (Table 4). Potentially contributing to this is the lower proportion of patients with SSR avid disease and the high proportion of tumours of unknown primary amongst our cohort compared with prior publications^6,8,9^. A previous series^8^ suggested that amongst G3 NEN, unknown primary was associated with poor prognosis (p = 0.04 in univariate analysis and p = 0.052 in multivariate analysis), which likely is influenced by the presence of stage IV disease in this population. Referral bias may also play a role; Queen Elizabeth Hospital is a tertiary referral centre for NET and therefore may manage a higher proportion of aggressive or treatment refractory cases. We postulate that the higher proportion of unknown primaries in our cohort compared with previous series^6,8,9^ could be in part, due to our status as a tertiary referral centre–such cases may be referred for specialized investigations to localize a primary tumour, or for management of advanced metastatic disease. Additionally, fasting gut hormones were not measured in 5/13 unknown primaries; it is possible that more complete biochemical workup may have yielded diagnosis of a pancreatic primary for some of these cases.

**Table 4:** Summary of previously published cohort studies including G3 NET.

First author, year	Population	SSR avidity	Ki67	Treatment	Disease control rate (PR + CR + SD)	Progression free survival in treatment group(s)	Median OS of NET G3 cohort
Our study	NET G3 n = 26	60% (n = 9)	Median 30%	SSA n = 9	SSA: 33% (3/9)	SSA: median 4 months	19 months
				Chemotherapy n = 11	Chemotherapy: 45% (5/11)	Chemotherapy: median 3 months	
				CAPTEM n = 8	CAPTEM: 50% (4/8)		
				Platinum based n = 3	Platinum: 33% (1/3 )		
Heetfeld, 2015^8^	GEP NET G3 n = 37	92% (n = 21)	Median 30%	Platinum-based chemotherapy n = 12	33% (n = 3)	Median PFS:2.4 months	99 months
Raj, 2017^9^	Pancreatic NET G3 n = 16	87% (n = 13)	Mean 47%	SSA n = 3	SSA: 33% (n = 1)	NR	52 months
				Platinum based chemotherapy n = 10	Platinum: 60% (n = 6)		
				Alkylating agents n = 12	Alkylating: 75% (n = 9)		
Velayoudom-Cephise, 2013^6^	Non-small cell thoracic NET G3 N = 12	88% (n = 7)	Median 21%	Platinum based chemotherapy n = 4	75% (n = 3)	NR	41 months
Sahu, 2019^13^	NET G3 n = 11	NR	NR	CAPTEM n = 11	NR	NR	19 months
Apostolidis, 2019^25^	NET G3 n = 89	NR	NR	Platinum based n = 34	Platinum: 70.6%	Platinum: 6.7 months	Not reached
				FOLFOX n = 17	FOLFOX: 82.4%	FOLFOX: 8.6 months,	
				Temozolomide based n = 12	Temozolomide: 58.3%	Temozolomide: 10.8 months	
				Other n = 16	Other: 62.5%	Other 12.0 months, *p* = *NS for platinum compared with other individual treatments*	
Hijioka, 2017^23^	Pancreatic NET G3 n = 21	NR	Median 28.5%	Chemotherapy n = 16	Platinum 37.5%	NR	42 months
				Platinum based n = 8	Everolimus 0%		
				Other n = 8	Gemcitabine 33%		
				Everolimus n = 3	Fluropyrimidine 0%		
				Gemcitabine based n = 3			
				Fluoropyrimidine n = 2			
Shibuya, 2018^24^	Pancreatic NET G3 n = 11	NR	NR	Streptozocin based n = 11	54.5%	NR	NR
Chan, 2019^15^ (Chan et al., unpublished observations)	G3 NET n = 64	NR	NR	CAPTEM	98%	NR	30 months
Rogowski, 2019^14^	G3 NET n = 20	NR	NR	CAPTEM n = 20	70%	15.3 months	22 months

SSA: somatostatin analog; PD: progressive disease, SD: stable disease; PR: partial response; OS: overall survival; PFS: progression free survival CAPTEM: capecitabine and temozolomide; GEP: gastroenterohepatic; NR: not reported; NS: non-significant.

Though long-acting SSA have been included in treatment algorithms for G3 NET^10^, evidence to support their efficacy in this population is limited. Raj et al.^9^ in a series of 21 patients with pancreatic G3 NET describe 3 cases treated with long acting SSA; 1 case showed SD for an unknown interval and the other two cases had PD. In our series, 70% of cases had PD following treatment with SSA, despite SRS avidity. However, the single case with carcinoid syndrome had improvement in symptoms despite demonstrating PD after 1 month, suggesting a potential role of SSA as an anti-secretory agent in this setting. Though select cases of G3NET may benefit from SSA treatment, evidence for their efficacy as anti-proliferative agents in this population is currently limited.

In our series, disease control was observed in only 1/3 patients treated with platinum-based chemotherapy. Given the lack of distinction of G3 NET from G3 NEC until recently, historically platinum-based chemotherapy has been considered first-line for these tumours^10,12^. Extrapolation from the NORDIC NEC study suggests that G3 NET may be less responsive to first-line (typically platinum-based) chemotherapy than G3 NEC, based on the lower response rates and shorter survival seen in patients with Ki67 < 55% compared with Ki67 ≥ 55%, however, the proportion of these cases which are truly G3 NET cannot be determined^12^. Disease control rates to platinum-based chemotherapy in retrospective cohorts for G3 NET range from 33 to 75% across published series^6,8–10,23,25^ (Table 4). Few series have reported progression free survival; in a previous series^8^ of 12 cases of G3 NET cases treated with platinum-based chemotherapy reported median progression free survival of 2.4 months, which is in keeping with PFS of 2 months observed in our platinum based chemotherapy subgroup. PFS of 6.4 months was reported by Apostolidis et al*.*^25^ for patients with pancreatic G3 NET treated with platinum based chemotherapy.

CAPTEM has shown efficacy in treatment of well-differentiated NET^26,27^; further investigation of the efficacy of CAPTEM specifically for G3 NET has ensued. Sahu et al.^13^ in a cohort of 32 cases including both G2 (n = 21) and G3 (n = 11) NET treated with CAPTEM reported a DCR of 62.5% however, the response rate specific for the G3 group was not reported. Rogowski et al.^14^ in a cohort including 20 patients with G3 NET all with Ki67 < 55% showed disease control rate of 70% and PFS of 15.3 months. Chan et al.^15^ recently reported a series including 64 G3 NET cases treated with temozolomide-based regimens (mostly CAPTEM) reported disease control of 98% (Chan et al., unpublished data) in the G3NET group.

In our series all cases which responded to CAPTEM had Ki67 of < 55%, further suggesting this regimen may have better efficacy for tumours with a lower proliferation rate within the G3 NET category. At our centre, decisions regarding chemotherapy regimen are made by the NET oncology team based on pathological features and clinical behavior. Our approach to treatment of this disease has evolved since 2012 as new evidence regarding the G3 NET classification has emerged. Based on the existing evidence^12^, platinum-based chemotherapy tends to be selected for more aggressive appearing tumours with rapid progression and higher Ki67**.** Given this, it is not practical for us to compare response rates of PFS between the two regimens in our cohort or to draw any conclusions about the efficacy of each regimen for G3 NET, as we believe patients in our cohort treated with platinum based chemotherapies have worse prognosis at baseline. A prospective, phase II randomized control trial comparing CAPTEM to platinum-based chemotherapy for G3 NEN is underway (ECOG-ACRIN EA2142) and results are awaited^28^.

The strengths of our study include that it is a large cohort of G3 NET treated at a single institution, and that we report on clinical outcomes for all patients including PFS, which is an important clinical outcome in the NET population. The main limitation of our study is its retrospective design which does not allow for comparison of different treatment strategies. Furthermore, the worse survival outcomes in our study compared with previous literature^5–8^ may limit the generalizability of our findings. Pathological diagnosis for majority of cases was based on tissue obtained from biopsy of liver metastases; we acknowledge that the grade of a single tissue sample obtained from biopsy may not reflect the grade of the primary or from other metastatic sites. It is therefore possible that some of our cases had other sites of poorly differentiated disease in keeping with NEC, which could have potentially contributed to our survival outcomes. Our sample size is an additional limitation. Though our cohort is large given the rarity of G3 NET, our analysis of 26 cases is susceptible to skew from individual cases, which may further limit the generalizability of our findings.

G3 NET are heterogenous group of tumours which present management challenges due to the lack of clinical evidence to guide treatment decisions. SSAs are indicated for patients with symptomatic carcinoid syndrome, which appears to be rare in this population. Select cases may show disease control following treatment with long acting SSA and/or chemotherapy. Evidence to stratify which cases will benefit from SSA or chemotherapy is currently lacking and therefore an important topic of future study. However, the efficacy of currently available treatments for G3 NET appears modest as evidenced by the short PFS seen in both treatment cohorts and therefore, more effective therapies are needed. Given the lack of high quality evidence to inform treatment, we suggest that management of G3 NET be individualized, and ideally carried out in association with centres of excellence with input from a specialist NET multidisciplinary team. While high quality randomized control trials in this population are clearly needed, real world observational studies are also valuable in furthering our understanding of this disease. In order to best inform future care, we suggest that multidisciplinary teams establish protocols for surveillance imaging and biochemistry (CgA and 5HIAA) following initiation of therapy. Given the rapid disease progression seen in several cases in our cohort, more frequent follow-up and surveillance imaging for patients with advanced disease at presentation may be warranted than with G1/G2 NET. For patients undergoing treatment with SSA and/or chemotherapy, we suggest performing surveillance imaging at intervals of three months or less, until disease trajectory is established (Supplementary Information).

## Supplementary Information

Supplementary Information.

## Data Availability

The datasets generated during and/or analysed during the current study are available from the corresponding author on reasonable request.
