# Supplementary figures and images for: Well-differentiated gastroenteropancreatic G3 NET: findings from a large single centre cohort
Source: Sci Rep. 2021 Sep 9;11:17947. doi: 10.1038/s41598-021-97247-x (PMC8429701; doi:10.1038/s41598-021-97247-x)

**Supplementary Figure I:** Survival for cases with Stage I-III vs. Stage IV disease

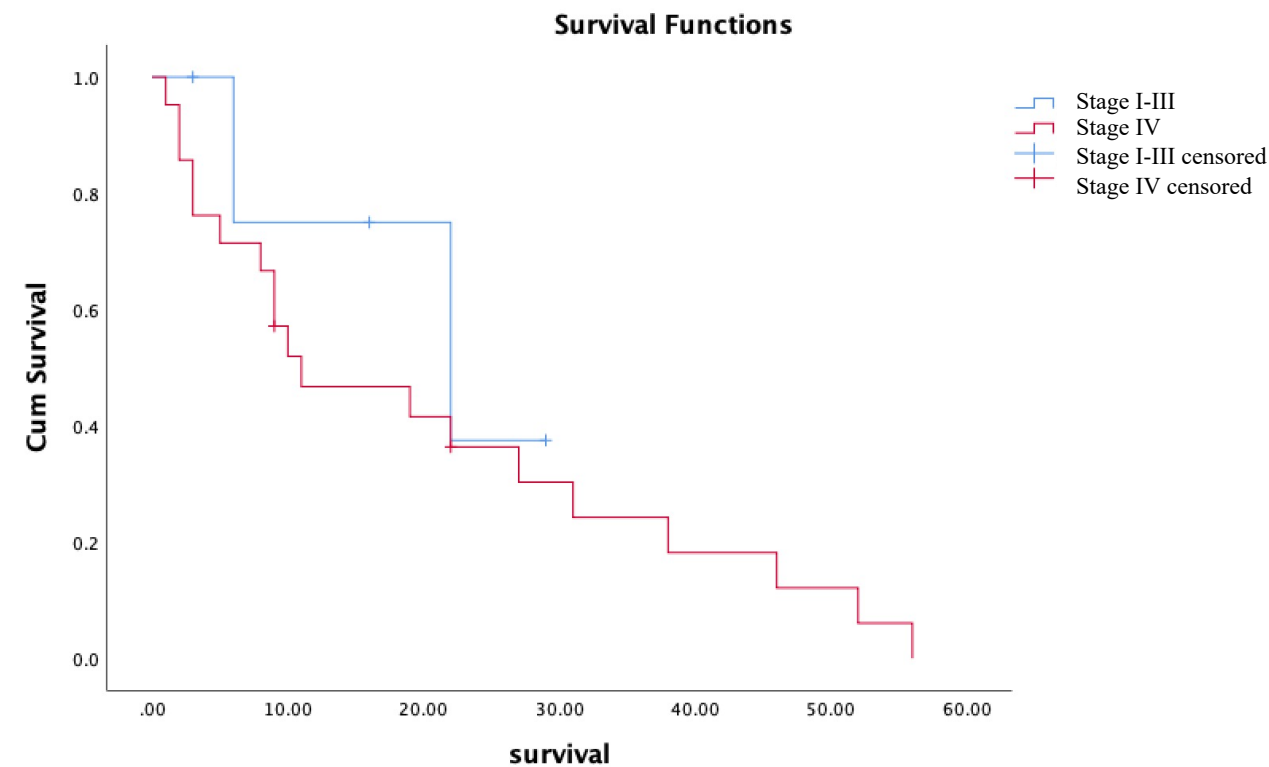

Supplement: Supplementary file 1 — Supplementary Information. [file 41598_2021_97247_MOESM1_ESM.pdf]
